# Pediatric Tuina for promoting growth and development of preterm infants: A protocol for the systematic review of randomized controlled trail

**DOI:** 10.1097/MD.0000000000010574

**Published:** 2018-05-04

**Authors:** Xinghe Zhang, Taipin Guo, Bowen Zhu, Qing Gao, Hourong Wang, Xiantao Tai, Fujie Jing

**Affiliations:** a School of Acupuncture—Tuina, Shandong University of Traditional Chinese Medicine, Jinan, Shandong; b School of Acupuncture—Tuina and Rehabilitation, Yunnan University of Traditional Chinese Medicine, Kunming, China.

**Keywords:** growth and development, infants, pediatric, preterm, protocol, systematic review, Tuina

## Abstract

**Background:**

Preterm infants are babies born alive before 37 weeks. Many survived infants concomitant with defects of growth and development, a lifetime of disability usually as following when insufficient intervention. In early intervention of preterm infants, pediatric Tuina shows good effect in many Chinese and some English clinical trials. This systematic review is aimed to evaluate the efficacy and safety of pediatric Tuina for promoting growth and development of preterm infants.

**Methods:**

The electronic databases of Cochrane Library, MEDLINE, EBASE, Web of Science, Springer, World Health Organization International Clinical Trials Registry Platform, China National Knowledge Infrastructure, Chinese Biomedical Literature Database, Wan-fang database, Chinese Scientific Journal Database, and other databases will be searched from establishment to April 1, 2018. All published randomized controlled trials (RCTs) about this topic will be included. Two independent researchers will operate article retrieval, screening, quality evaluation, and data analyses by Review Manager (V.5.3.5). Meta-analyses, subgroup analysis, and/or descriptive analysis will be performed based on included data conditions.

**Results:**

High-quality synthesis and/or descriptive analysis of current evidence will be provided from weight increase, motor development, neuropsychological development, length of stay, days of weight recovery to birthweight, days on supplemental oxygen, daily sleep duration, and side effects.

**Conclusion:**

This study will provide the evidence of whether pediatric Tuina is an effective early intervention for preterm infants.

**Ethics and dissemination:**

There is no requirement of ethical approval and informed consent, and it will be in print or published by electronic copies.

**Trail registration number:**

This systematic review protocol has been registered in the PROSPERO network (No. CRD42018090563).

## 1 Introduction

### 1.1 Description of the condition

Preterm is defined by WHO as babies born alive before 37 weeks.^[1]^ Every year, approximately 15 million babies are born too early, which means >1 in 10 babies.^[2]^ In Africa and South Asia >60% of international preterm births are occupied. Affected by the economics, preterm birth rate of lower income countries is 12%, while higher income countries are 9%. According to reliable trend preterm birth rates of 65 countries, all but 3 countries indicate an increase over the past 20 years.^[1,3]^ Preterm infants are prone to serious illness or even death, nearly 1 million preterm infants died each year, and be the second leading causes of deaths among children under the age of 5. Many survived infants’ body are immature because of the incompletion of pregnancy, thus these infants usually have defects of growth and development. If the defects cannot be remedied, these infants will face a risk of disability, including learning disabilities, dyskinesia, heart and other diseases.^[4–7]^ Study reported that the cost was $2568 in the first 2 years of preterm infants, while the term infants were $1285 in Canada,^[8]^ which means more than double costs will be taken from family.

### 1.2 Description and function of intervention

Pediatric Tuina is a unique massage therapy of traditional Chinese medicine (TCM). Based on children meridian and acupoint theory, which is different from standard human acupoint, most meridians and acupoints of children converge on hand and upper limb, such as spleen meridian, liver meridian, Banmen, Bagua, Neilaogong, Sanguan, and Liufu. While some acupoints are same, such as Pishu (BL20) and Guanyuan (CV4). The manipulation is also different, such as pushing spleen meridian, rubbing abdomen, transiting Neibagua, and stroking Qianxin (anterior fontanelle) characterized by light, fast, and soft. Clinical articles have been showed that pediatric Tuina has good effects on breathing, weight growth, peripheral perfusion, and infantile food accumulation.^[9–11]^ Study on premature infants with brain injury indicated that pediatric Tuina could improve the intelligence development and lower the incidence of cerebral palsy.^[12]^ In another study of heel lancing operation for preterm infants, acupressure at Kunlun (UB60) and Taixi (K3) could shorten procedural times and duration of crying.^[13]^

### 1.3 Why the review is important

Early intervention in neonatal intensive care unit (NICU) and post-hospital improve the growth and development, providing preterm infants from many complications. With early intervention, preterm infants are at low risk of disability, and have favorable prognosis.^[14–16]^ As an early intervention of TCM, pediatric Tuina has been used in NICU and post-hospital. Some clinical trials reported that there are effects of pediatric Tuina on preterm infants.^[17,18]^ However, some nonstandard measurement, nonuniformed outcomes, and other factors of literature limited the evidence. In order to testify the efficacy and safety of pediatric Tuina for promoting growth and development of preterm infants, the evidence-based review is necessary. However, no review or protocol about this topic has been published. So, this review is urgently needed to accomplish.

## 2 Methods

This systematic review protocol has been registered in the PROSPERO network (No. CRD42018090563). Systematic review will be performed according to Cochrane Handbook 5.2.0.

### 2.1 Selection criteria

#### 2.1.1 Types of studies

Randomized controlled trial (RCT) and blinded research will be included. Published clinical trials that reported the efficacy and safety on pediatric Tuina for promoting growth and development of preterm infants will be included. RCTs that involve at least 1 pediatric Tuina related treatment to preterm infants, and 1 control treatment (or blank treatment) will be included. As there is a risk of interference with the outcome, nonrandomized controlled trials will be excluded. Studies of animal experiment, review, case report, and discussion will be excluded.

#### 2.1.2 Types of patients

Infants meet the diagnostic criteria of preterm that are defined by WHO (babies born alive before 37 weeks), without limits on gender, race, nationality, and medical units.

#### 2.1.3 Types of interventions and comparisons

Intervention can be any type of pediatric Tuina: acupressure, pushing, rubbing, transiting, stroking, and chiropractics based on meridian-acupoint theory of children. Multiple control interventions will be included: no treatment, placebo and other interventions (e.g, acupuncture, moxibustion, drugs, physical interventions, gentle touch, and other massage therapies). Comparisons contain pediatric Tuina and its relation will be excluded. Interventions of pediatric Tuina combined with other therapies will also be included, only if these combinations are compared to the other therapies semplice.

#### 2.1.4 Types of outcomes

Primary outcomes of review include weight increase, motor development, neuropsychological development, and side effects. Secondary outcomes include length of stay, days of weight recovery to birthweight, days on supplemental oxygen, and daily sleep duration.

### 2.2 Search methods for identification of studies

#### 2.2.1 Electronic searches

The primary electronic databases include: Cochrane Library, MEDLINE, EBASE, Web of Science, Springer, World Health Organization International Clinical Trials Registry Platform (ICTRP), China National Knowledge Infrastructure (CNKI), Wan-fang database, Chinese Scientific Journal Database (VIP), Chinese Biomedical Literature Database (CBM), and other databases. All published RCTs about this topic from establishment to April 2018 will be included.

Exemplary search strategy of MEDLINE is listed in Table 1, terms are conform to medical subject heading (MeSH). According to the difference of databases, keywords may combined with free words and comprehensive search will be performed.

**Table 1:**
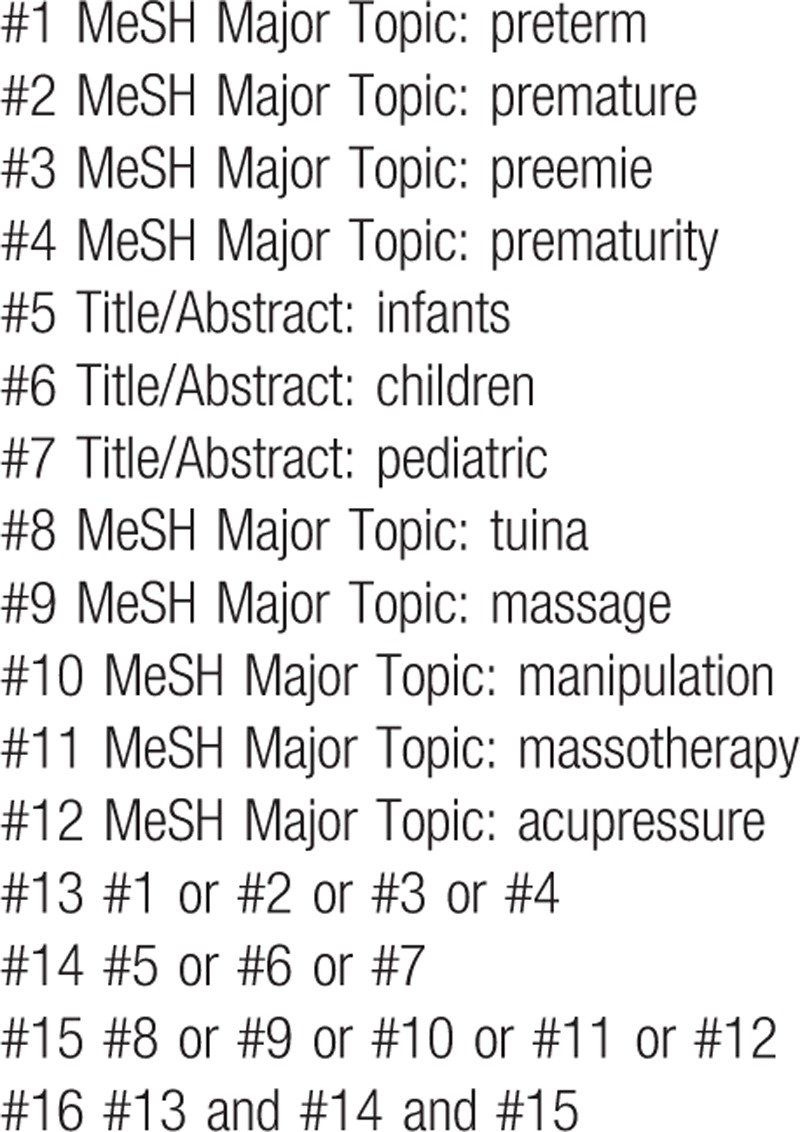
MEDLINE search strategy.

### 2.3 Data collection and analysis

#### 2.3.1 Selection of studies

**Figure 1:**
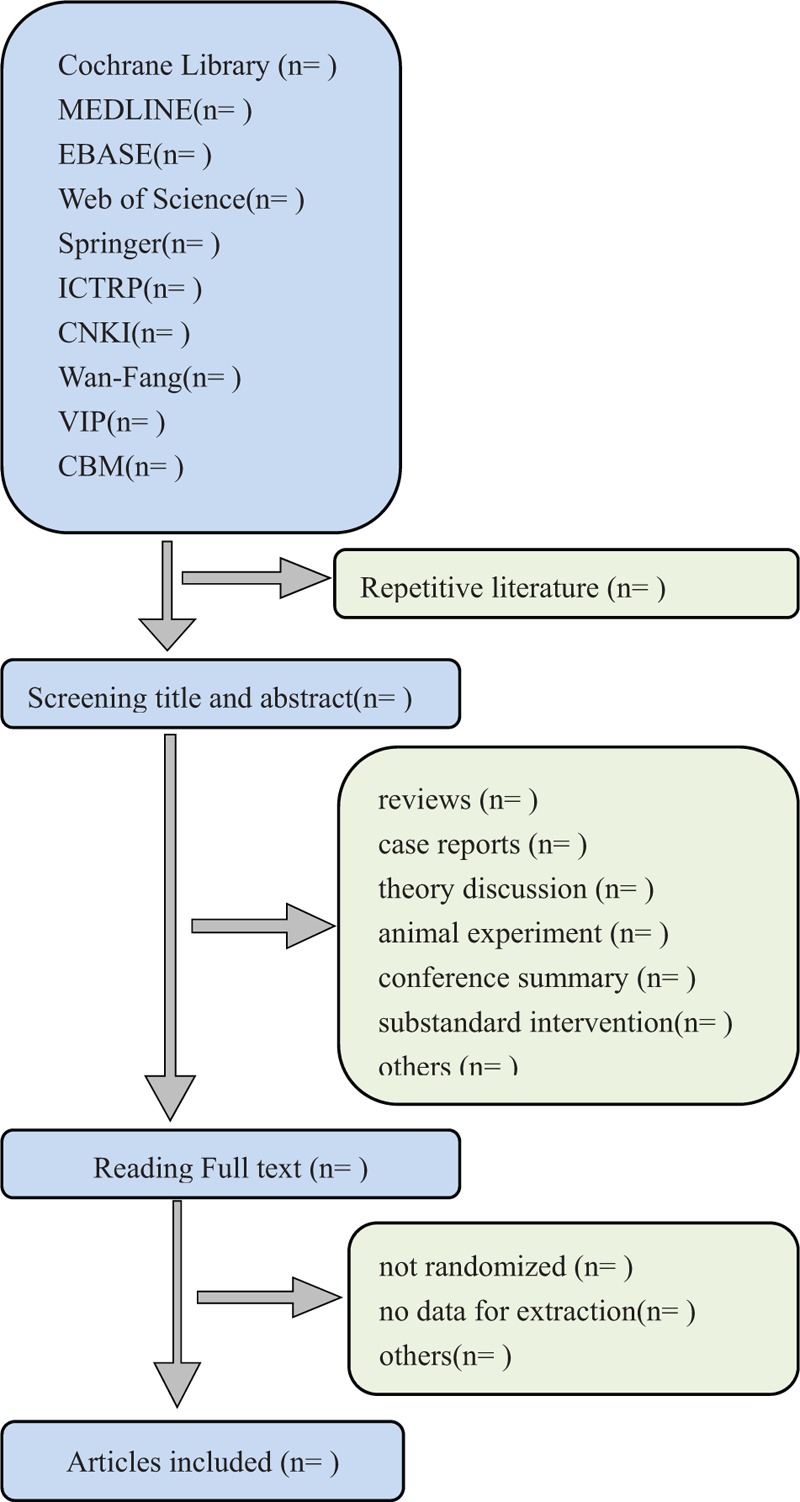
Flow diagram of studies identified.

Two authors (XHZ and BWZ) will select clinical trials depending on inclusion criteria. After the title and abstract are screened, literatures that are not related and do not meet the criteria will be excluded. Screening operation will flow the diagram of Figure 1. If the full literatures are unable to obtained or related data is incomplete, we will contact the corresponding author. If the selection being divergence, third-party experts will be consulted for resolution.

#### 2.3.2 Assessment and quality of included studies

Two authors (TPG and HRW) will evaluate quality of included articles and assess the risk of bias based on Cochrane Handbook 5.2.0. Quality assessment of included studies contains randomized method, allocation concealment, blinding of participants and personnel, blinding of outcome assessment, completeness of outcome data, and selective reporting. Divergence of evaluation will also consult third-party experts.

#### 2.3.3 Data extraction

Two authors (BWZ and QG) will extract data of included studies independently. The basic information and other data will be extracted and compiled to the standardized form: title (numbering, first author's name, and publication date), participants (number, subgroup, gender, and age), intervention (method, time, and cycle), outcome (weight gain, motor development, neuropsychological development, length of stay, days of weight recovery to birthweight, days on supplemental oxygen, daily sleep duration, and side effects).

#### 2.3.4 Measures of treatment effect

Two authors (XHZ and TPG) will perform analysis independently and then cross-check treatment effect with Review Manager 5.3.5. Dichotomous data will be presented by risk ratio (RR) with 95% confidence intervals (CIs). Continuous data will be presented by mean difference (MD) or standard mean difference (SMD) with 95% CI. Other binary data will be changed into the RR form for analysis.

#### 2.3.5 Dealing with missing data

As there is possibility of missing data in literatures, we will contact the corresponding authors by email or other contacts. If the missing data are unavailable, we will analysis the existing data that is supposed as random missing.

#### 2.3.6 Assessment of heterogeneity

The heterogeneity of studies will be evaluated by *Q*-test and *I*^*2*^ statistic with RevMan5.3.5. The following criteria will be used: *I*^*2*^ < 50% will be deemed as low heterogeneity; *I*^*2*^ between 50% and 75% will be considered as moderate heterogeneity; *I*^*2*^ > 75% will be considered as high heterogeneity.

#### 2.3.7 Assessment of reporting bias

Publication bias and other reporting biases will be assessed by creating funnel plots. Symmetric funnel plots indicate low risk of bias, while dissymmetry ones may indicate high risk.

#### 2.3.8 Data synthesis

A meta-analysis or descriptive analysis will be performed, based on the intervention methods, the measurement methods, and heterogeneity levels, etc. If clinical and methodological heterogeneity are low, the fixed-effect model will be applied by merger analysis; the random-effects model will be applied by merger analysis when heterogeneity indicates a moderate level. If, however, a significant level of heterogeneity is found, a descriptive analysis will be performed instead.

#### 2.3.9 Subgroup analysis

Subgroup analysis will be performed based on the findings from the data synthesis, and if the heterogeneity is found to have been caused by particular features of the included studies (e.g, the intervention methods [type, time, and cycle] and the measurement methods used in the clinical trials), subgroup analysis will be conducted relevant to these categories.

## 3 Discussion

Pediatric Tuina is a treatment originated from ancient China, which is an essential part of children's disease treatment in history. With the features of simpleness, high performing and cost-effective, pediatric Tuina is widely used in ancient time and nowadays. As the preterm birth rate is increasing^[1]^ and the recognition of pediatric Tuina, it is increasingly applied to early intervention of preterm infants.

Up to now, many related clinical trials were published but high quality trail is still lack. This review will begin when necessary trails are meeting. In order to give compelling evidence and better guide in clinic practice, all actions of this review will be performed according to Cochrane Handbook 5.2.0.

## Author contributions

**Conceptualization:** Hourong Wang, Xinghe Zhang.

**Data curation:** Qing Gao, Bowen Zhu.

**Investigation:** Fujie Jing.

**Methodology:** Xinghe Zhang, Xiantao Tai, Fujie Jing.

**Validation:** Xiantao Tai.

**Visualization:** Xiantao Tai.

**Writing – original draft:** Xinghe Zhang, Tainpin Guo.

**Writing – review & editing:** Tainpin Guo, Bowen Zhu.
